# The study on the role of dedicators on promoting cooperation in public goods game

**DOI:** 10.1371/journal.pone.0257475

**Published:** 2021-09-20

**Authors:** Zhenghong Wu, Huan Huang, Qinghu Liao

**Affiliations:** 1 College of Economics and Management, Civil Aviation University of China, Tianjin, China; 2 School of Public Management, Tianjin University of Commerce, Tianjin, China; Kyushu Daigaku, JAPAN

## Abstract

In daily life, some people are always seen dedicating available resources to support collective activities. In this paper, we call these people who care group goals more than individual goals dedicators. Inspired by this phenomenon, we studied the role of dedicators on the evolution of cooperation in public goods game (PGG) based on a Chinese Folk Spring Festival Gala. Three types of agents were introduced into our PGG model including cooperators, defectors and dedicators. Dedicators tried to donate when the Gala was short of funds. Cooperators and defectors imitated the strategy of the highest-payoff neighbor based on the rational mechanism. And their imitating probability was modified on account of the emotional mechanism for positive effect of the dedicator’s donating behavior and negative effect of continuous poor performance. Through numerical simulations, we found that the existence of dedicators can indeed promote cooperation in PGG. It should be noted that dedicators’ willingness to donate was more important than their donation quantity in facilitating cooperation. And the stronger the emotional effect intensity of dedicators’ donating behavior was, the better. So, the selfless dedication of participants should be praised to promote cooperation by improving their emotional effect intensity. Last but not least, a reasonable activity budget was needed to sustain the highest level of cooperation.

## Introduction

Evolutionary game theory is about unraveling the mysteries of cooperation in biological and social systems [1]. A focus of this theory is the situation known as social dilemmas, where short-term self-interest conflicts with long-term group interest [2]. Recently, it is theoretically proved that there is a common dilemma structure mainly observed in binary game by introducing the concept of universal dilemma strength [3–7]. Different from this special case, however, most real-world dilemmas violate the binary format and extend to the closely related public goods game.

Most interactions in human social networks are simulated in multiplayer games. There are significant social dilemmas of multiplayer game in China, which need to be solved urgently. The Folk Spring Festival Galas have sprung up in some villages in Zhejiang province of China in the last few years. These folk activities are always conducted by village heads. In addition to the funding from local governments and companies, rest of the funds are raised by villagers’ voluntary contribution. Everyone can participate in this gala, regardless of donating or not. This activity enriches the cultural life of local villagers, but it is facing a pressing dilemma. Due to the shortage of funds, many villages have stopped hosting the gala. So, how to sustain this folk cultural activity is an issue that need to be solved by the organizer urgently. Different from others, Yunlin Gala is one of the few exceptions, which has been held for ten years in Yunlin village. Although facing the same problem of fund shortage, it is held successfully every year. Inspired by the fact, we try to investigate the reasons for success of this case to find a solution to solve the thorny problem of other villages. After some comparisons, we find that besides the village head there exist dedicators such as college students, art lovers and so on show selfless devotion to conduct Yunlin Gala. Unlike cooperators they concern group goals more than personal goals, so we call them dedicators. Therefore, we have some deep thinking: Are these dedicators the key to promote cooperation in Yunlin Gala? If yes, how can they make it? And are they the more the better? To get the answer of those meaningful questions, we start our work.

In view of natural selection everyone is a competitor to others. It is hard to stop making trouble to each other, let alone cooperate. However, cooperation is widespread around us in real life. Therefore, many scholars devote themselves to explaining this ubiquitous but inconceivable phenomenon in animal and human societies [8–11]. As an outstanding representative of evolutionary games, PGG provides a basic framework to research the origin and stability of cooperation [12]. In a standard PGG, each cooperator contributes *c* to the common pool, but defectors not. Then total contributions are equally divided among all agents after multiplied by a synergy factor *r*. It is apparently that defection is the optimal strategy, cooperation becomes a social dilemma. The Folk Spring Festival Gala is similar to the framework of PGG, thus we study the role of dedicators on cooperation on the basis of this model.

To explore the emergence and maintenance of cooperation, several mechanisms have been proposed by PGG, such as heterogeneity [13–16], indirect reciprocity [17–24], social diversity [25–28], punishment [29–39], reward [38–50], reputation [51–57], voluntary contribution [58–62], population structure [63–67] and so on. Recently, some scholars put forward that emotions also have a significant effect on the origin and stability of cooperation [68–72]. Every mechanism is identified through a spatial structure, which stems from the pioneering work of Nowak and May [12]. Because compared to the regular lattice, agents have different number of neighbors in a spatial structure which is much more accordance with the real world.

A few researchers have put forward that some factors can influence the evolution of cooperation in Chinese traditional public cultural activity based on PGG, such as publishing the donation list [73–75], donating time [76], leadership by example [77] and people who donate more money [78]. The above researches mainly assume that agents only focused on personal goals. Different from them, in this paper we study the role of dedicators who pay more attention to the group goals in addition to personal goals. Compared with others, the biggest feature of Yunlin Gala is the existence of those dedicators. Every winter vacation the local college students will come back to help the village head organize and advertise the gala. Art lovers are responsible for programming performances. And when the funds do not reach the budget, they are likely to give money to support the gala. Players are driven by “rationality” to make better decisions and maximize their personal payoff [79]. But human beings are not only rational, but also emotional. Emotions have been demonstrated can affect decision-making [68–71]. So we assume that some agents would be moved by dedicators’ donating behavior to be cooperators. However, it should be noted that dedicators’ continuous willingness to donate is not strong. And the amount of money a dedicator can donate is limited. What’s more, successive poor performances will discourage donating among people. Summarily, we focus on exploring the role of dedicators on the emergence and maintenance of cooperation in the above condition based on PGG.

The rest of this paper is structured as follows. In Section 2, our PGG model is described. In Section 3, we present the numerical simulation results and analysis. In Section 4, conclusions are proposed according to the experimental results.

## Model and methods

Multiplayers’ interaction is not as simple as the relationship between two, is interdependent and entangled. Unlike the 2 × 2 game, secondary and direct neighbors in the multiplayer simulation can affect the payoff of the focus agency [80]. To explain the influence of dedicators, three types of agents are classified in a spatial structure, covering cooperators, defectors and dedicators. Cooperators and defectors only choose to donate money or not. Normally, different from cooperators and defectors, dedicators make non-monetary contributions to the Gala, but when lack of funds, they may donate money as much as they can.

Our PGG model is performed on a scale-free network. A randomly selected agent *i* has an own number of neighbors (*k*_*i*_) and attends *k*_*i*_ + 1 PGG groups. At the initial time step, the fraction of cooperators and defectors is equal. The fraction of dedicators (*f*_1_) is dynamic to satisfy experiment demand. The payoff obtained by each agent can be calculated as follows:

$$\begin{array}{c} P_{i}=\sum_{j\in \Omega_{i}}P_{i}^{j}=\sum_{j\in \Omega_{i}}\left(r\frac{n_{j}}{k_{j}+1}-c_{i}\right) \end{array}$$ (1)

where *P*_*i*_ denotes the payoff of agent *i*. Ω_*i*_ stands for the set of groups where *i* attends. *j* is one group of Ω_*i*_. *r* (> 1) is the game parameter indicating dilemma weakness [80], called synergy factor, reflecting the synergetic effects of cooperation. *n*_*j*_ represents contributions of group *j*. *k*_*j*_ is the number of neighbors of agent *i* in group *j*. *c*_*i*_ is contributions of agent *i*. If agent i is a cooperator, *c*_*i*_ = 1; if agent i is a defector, *c*_*i*_ = 0; otherwise, *c*_*i*_ = *c*_*d*_ (*c*_*d*_ ≥ 0, *c*_*d*_ will be explained detailly in the following paragraph).

**Fig 1:**
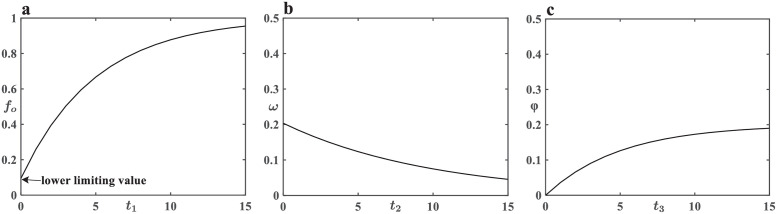
Descriptions of relationships between some parameters. Here *θ*_1_ = 0.2, *δ* = 0.5, *θ*_2_ = 0.2, *θ*_3_ = 0.2. (a) The dedicator’s probability of donating (*f*_*o*_) for different break time steps (*t*_1_) when *Ti* < *Tr* has ever happened. (b) The extra cooperation probability (*ω*) for different time steps (*t*_2_) after dedicators’ donating behavior. (c) The extra defection probability (*ϕ*) for different successive times of poor performances (*t*_3_).

It is assumed that the Folk Spring Festival Galas can be held as required if total group contributions (*Ti*) will reach budget (*Tr*), otherwise, the quality of the activity will decline. In real social life, if an activity fails continuously, people will have less confidence in its success and may not choose to support it. It means the dedicator is more willing to donate discontinuously instead of continuously. So, we assume that when *Ti* < *Tr* happens for the first time, dedicator’s probability of donating *f*_*o*_ will be equal to 1(see the upper formula of Eq (2)). When *Ti* < *Tr* has ever happened, *f*_*o*_ will be an increasing function of break time steps (*t*_1_) (see the lower formula of Eq (2)). We define dedicator’s probability of donating as follows:

$$\begin{array}{c} f_{o}=\{\begin{array}{ccc} 1 & , & Ti<Tr\;\;\;happens\;first\;time\\ 1-exp(-0.1-\theta_{1}t_{1}) & , & Ti<Tr\;\;has\;\;ever\;happened \end{array} \end{array}$$ (2)

where *f*_*o*_ is the dedicator’s probability of donating as *Ti* < *Tr*, 0 < *f*_*o*_ ≤ 1. *t*_1_ is break time steps, indicating the continuous time steps in which contributions reach budget after the last occurrence of *Ti* < *Tr*. If donating behavior happened among dedicators in last step (*t*_1_ = 0), *f*_*o*_ will decrease to a lower limiting value (Fig 1a). However, due to the prosocial preference of human beings, *f*_*o*_ is always greater than zero. We adopt an exponential probabilistic model to describe the relation between *f*_*o*_ and *t*_1_. *θ*_1_ is the recovering coefficient, which reflects the recovery speed of the dedicator’s willingness to donate. The greater *θ*_1_ is, the more rapidly *f*_*o*_ enlarges.

For rational consideration of survival, there exists a donation threshold λ for each dedicator, which indicates the maximum amount of money a dedicator can afford to give. Therefore, dedicator’s monetary contributions *c*_*d*_ is calculated as follows:

$$\begin{array}{c} c_{d}=\{\begin{array}{ccc} \min(\frac{Tr-Ti}{f_{1}N},\lambda ) & , & t_{1}=0\;\\ 0 & , & t_{1}\neq 0\; \end{array} \end{array}$$ (3)

where *Tr* − *Ti* represents the shortfall between the funds needed for the Gala and donations of cooperators in this generation. The shortfall of money will be shared equally by the dedicators. *N* is the total number of people. *f*_1_ denotes the proportion of dedicators. λ represents the donation threshold of each dedicator. When dedicators needn’t donate continuously (*t*_1_ = 0), they will donate as much as they can. Otherwise, they will not. To show the model briefly, we assume that any dedicator has the same value of λ and *c*_*d*_.

**Fig 2:**
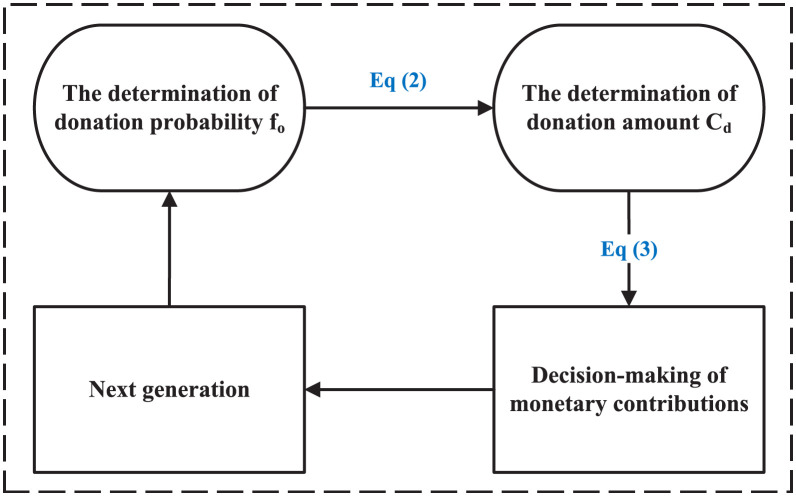
The circulatory process of decision-making of dedicators. Dedicators’ contributions include monetary and non-monetary contributions. Decision-making of monetary contributions requires determining in advance the possibility and amount of donations.

Contributions of dedicators include monetary and non-monetary contributions. The decision process for a dedicator’s monetary contributions is shown in Fig 2. When the contributions are less than budget, dedicator’s monetary donation decisions are influenced by both collective and individual goals based on Eqs (2) and (3). The decision of a dedicator’s monetary contributions is divided into two parts: the determination of whether to donate (with the probability *f*_*o*_) and the question of how much to donate. The last step of this generation is the first step of the next generation.

Normally, cooperators and defectors are rational payoff-driven agents who always change strategies to maximize their payoff. They make the initial decision by imitating the strategy of the neighbor who has highest payoff (excluding dedicators) with the following probability:

$$\begin{array}{c} W^{\prime }\left(s_{i}\leftarrow s_{j}\right)=\frac{1}{1+exp[\left(P_{i}-P_{j}\right)/\phi ]} \end{array}$$ (4)

where *ϕ* represents the amplitude of environment noise [61, 81]. *s*_*i*_ and *s*_*j*_ denote the strategies of agent *i* and neighbor *j*, respectively. *P*_*i*_ − *P*_*j*_ is the difference of payoff between agent *i* and neighbor *j*.

However, human-beings can’t be completely rational. Sometimes, due to emotion mechanism, decisions of cooperators and defectors will be influenced by donating behavior of dedicators inevitably. Thus, an extra cooperation probability *ω* is added to cooperators and defectors. On the basis of common sense, we assume that the extra cooperation probability *ω* is a decreasing function of time steps. An exponential probabilistic model is chosen to describe the variation trend of *ω* (Fig 1b). So, the extra cooperation probability is expressed by the following function:

$$\begin{array}{c} \omega =\frac{\delta }{3\exp[\theta_{2}\left(0.5t_{2}-1\right)]} \end{array}$$ (5)

where *ω* denotes the extra cooperation probability, 0 < *ω* ≤ 0.2. *t*_2_ denotes the successive time steps after dedicators’ donating behavior. It should be noted that we only consider the single impact of the time-closest donating behavior instead of the accumulated impact of all donating behavior. *δ* is emotion coefficient, which represents the emotional effect intensity of dedicators’ donating behavior. *θ*_2_ is a positive sustained coefficient, which affects the reduction velocity of emotional effect of dedicators’ donating behavior. The greater *θ*_2_ is, the more slowly *ω* declines.

When total contributions are still less than the budget after dedicators donating money, the quality of Spring Festival Gala will decline. Audiences can tolerate the poor performance one time, but cannot endure continuously. So, successive poor performances will add an extra defection probability *φ* to cooperators and defectors. Therefore, we choose an exponential probabilistic model to describe the variation trend of *φ* (Fig 1c). In addition, this model can show our assumption that the extra defection probability will grow more and more slowly with the increase of *t*_3_. So, we define the extra defection probability as follows:

$$\begin{array}{c} \varphi =[1-\exp\left(-\theta_{3}t_{3}\right)]/5 \end{array}$$ (6)

where *φ* is the extra defection probability, 0 < *φ* ≤ 0.2. *t*_3_ denotes the successive times of poor performances. *θ*_3_ is the negative sustained coefficient, positively correlated with *φ*, which reflects the sustained impact of the poor performance. All parameters in this paper are defined and explained in Table 1.

**Table 1:** The definitions and descriptions of parameters.

Parameter	Definition and description
P	The payoff of the agent
Ω	The set of PGG groups where the agent attends
r	The synergy factor
c	The contributions of the agent
Ti	The contributions donated by all of cooperators
Tr	The budget of the gala
λ	The donation threshold of dedicators
N	The population
*f*_1_	The fraction of dedicators
*f*_2_	The fraction of cooperators
*f*_3_	The fraction of defectors
*f*_*o*_	The dedicators’ probability of donating when *Ti* < *Tr*
*θ*_1_	The recovering coefficient, the recovery speed of dedicators’ willingness to donate
*θ*_2_	The positive sustained coefficient, which affects the reduction velocity of emotional effect of dedicators’ donating behavior
*θ*_3_	The negative sustained coefficient, the sustained impact of the poor performance
*ϕ*	The amplitude of environment noise
*ω*	The general extra cooperation probability
*δ*	The emotion coefficient, the emotional effect intensity of dedicators’ donating behavior
*φ*	The general extra defection probability
*t*_1_	Break time steps, which means cooperators’ contributions reach budget in the continuous time steps after latest condition of *Ti* < *Tr*
*t*_2_	The time steps after dedicators’ donating behavior
*t*_3_	The successive times of poor performances

Considering emotional factors, the imitation probability, quantified by PW-Fermi rule, can be modified [82]. Cooperators and defectors update strategies with the modified probability:

$$\begin{array}{c} W\left(s_{i}\leftarrow s_{j}\right)=\{\begin{array}{c} \frac{1}{1+\exp[\left(P_{i}-P_{j}\right)/\phi }+\omega -\varphi \;\;\;\;,\;\;\;c_{j}=1\\ \frac{1}{1+\exp[\left(P_{i}-P_{j}\right)/\phi }-\omega +\varphi \;\;\;\;,\;\;\;c_{j}=0 \end{array} \end{array}$$ (7)

where *c*_*j*_ = 1 means the strategy of neighbor *j* is cooperation in last round, and *c*_*j*_ = 0 means defection. If the selected neighbor *j* is a cooperator, the transition probability *W*(*s*_*i*_ ← *s*_*j*_) updates based on the upper formula. Conversely, *W*(*s*_*i*_ ← *s*_*j*_) changes according to the lower formula.

**Fig 3:**
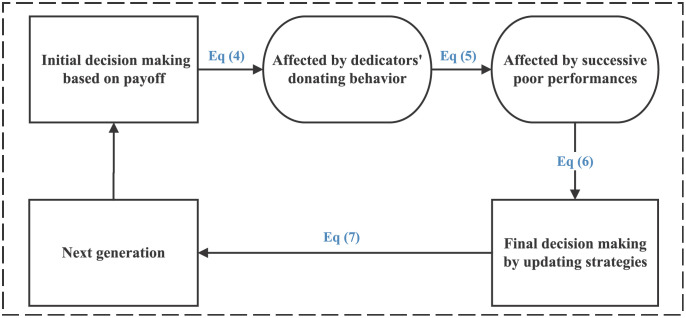
The circulatory process of decision-making of cooperators and defectors. Cooperators and defectors make decisions at the same time. Decisions of cooperators and defectors are driven by personal payoff and affected by dedicators’ donating behavior based on emotional effect and successive poor performances.

The decision-making process of cooperators and defectors is displayed in Fig 3. As rational men, cooperators and defectors employ the same set of decision-making process. First of all, driven by personal payoff, the agent make the initial decision by imitating his/her neighbor’s strategy with the probability of *W*′(*s*_*i*_ ← *s*_*j*_). After that the agent will be affected by dedicators’ donating behavior and successive poor performances and update the strategy with the probability of *W*(*s*_*i*_ ← *s*_*j*_) to make the final decision.

## Simulation and analysis

To investigate the role of dedicators on the evolution of cooperation in the Folk Spring Festival Gala, a large number of simulations are performed in PGG model. The results are obtained by average over 30 independent runs for 10000 time steps. Each equilibrium datum is obtained by averaging the last 5000 generations of 30 independent runs. Initially, the population *N* = 1000, average number of neighborhood *k* = 4, budget *Tr* = 500, synergy factor *r* = 1.2, noise figure *φ* = 0.1, dedicators’ donation threshold λ = 0.5. The fraction of dedicators, cooperators and defectors is *f*_1_ = 0.1, *f*_2_ = 0.45, *f*_3_ = 0.45, respectively. The rest parameters are *θ*_1_ = 0.2, *δ* = 0.5, *θ*_2_ = 0.2, *θ*_3_ = 0.2. While one of the parameters changes, others keep stationary. In this paper, *ρ* refers to cooperation frequency which means the ratio of cooperators to the sum of cooperators and defectors. *Ts* represents the total contributions.

**Fig 4:**
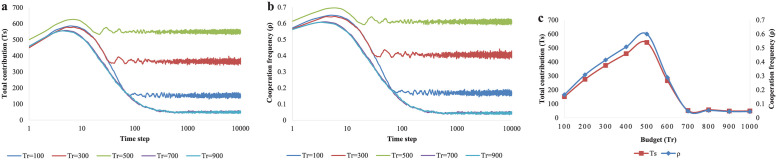
The evolution results of total contributions *Ts* and cooperation frequency *ρ* for different values of budget *Tr*. (a) The evolution process of total contributions for different budgets (*Tr* = 100, 300, 500, 700, 900). (b) The evolution results of cooperation frequency for different budgets (*Tr* = 100, 300, 500, 700, 900). (c) The equilibrium results of total contributions and cooperation frequency for different budgets from 100 to 1000 with an interval of 100.

Firstly, the role of budget (*Tr*) is discussed in Fig 4. We can find from the figure that there is a threshold (about 500 here) of *Tr*, which includes two meanings: (i) When *Tr* is not greater than the threshold, the equilibrium results of total contributions *Ts* can meet the required level (*Tr*) and the Gala will succeed. When *Tr* is greater than the threshold, the equilibrium results of *Ts* can’t reach *Tr* and the activities can not be held with high quality. (ii) When *Tr* is less than the threshold, the equilibrium results of *Ts* and *ρ* get larger as the threshold enlarges. Otherwise, *Ts* and *ρ* go down as *Tr* increases. As we can see, a too high budget will lead to a collapse of the whole cooperation system. Therefore, a reasonable activity budget needs to be decided by organizers to sustain the maximum level of cooperation.

**Fig 5:**
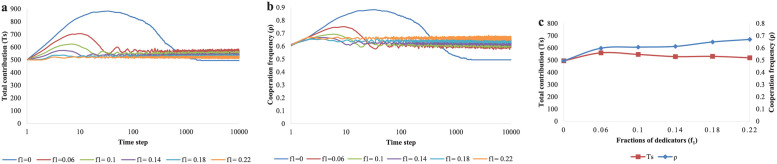
The evolution results of total contributions *Ts* and cooperation frequency *ρ* for different fractions of dedicators *f*_1_. (a) The evolution process of total contributions for different fractions of dedicators (*f*_1_ = 0, 0.06, 0.1, 0.14, 0.18, 0.22). (b) The evolution results of cooperation frequency for different fractions of dedicators (*f*_1_ = 0, 0.06, 0.1, 0.14, 0.18, 0.22). (c) The equilibrium results of total contributions and cooperation frequency for different fractions of dedicators (*f*_1_ = 0, 0.06, 0.1, 0.14, 0.18, 0.22).

Then, the impact of the fraction of dedicators (*f*_1_) on cooperation will be investigated. In real life, there are only a few dedicators. Therefore, the initial value of *f*_1_ is set to 0.1, and the research range of *f*_1_ is from 0 to 0.22. It can be drawn that the existence of dedicators can help total contributions exceed the budget and stabilize cooperation at high level. In Fig 5a and 5b, we can see that as *f*_1_ increases, the corresponding curves reach cooperation equilibrium more quickly and stably. Except for the dark blue curve, the other curves remain at higher level. It means dedicators can strongly stabilize and promote cooperation. Similar to Fig 5a and 5b, it can be seen that the red and blue curves are at lowest level when *f*_1_ = 0 in Fig 5c. Moreover, we find an interesting phenomenon. Under the condition of *f*_1_ > 0.06, the blue curve and the red curve diverge in Fig 5c. Through analyzing, we can speculate that: (i) *f*_1_ is directly proportional to *ρ* because more defectors will become cooperators with the increase of dedicators. (ii) *f*_1_ is inversely proportional to *Ts* as dedicators don’t need to donate after cooperators’ contributions reach the budget even though there are more dedicators.

**Fig 6:**
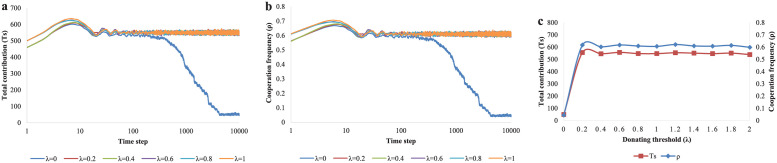
The evolution results of total contributions *Ts* and cooperation frequency *ρ* for different values of the donation threshold λ. (a) The evolution process of total contributions for different values of donation threshold (λ = 0,0.2,0.4,0.6,0.8,1). (b)The evolution results of cooperation frequency for different values of donation threshold (λ = 0,0.2,0.4,0.6,0.8,1). (c) The equilibrium results of total contributions and cooperation frequency for different values of donation threshold λ varying from 0 to 2 with an interval of 0.2.

Next, we will look at the effect of the donation threshold of dedicators (λ). We find that dedicators’ donating behavior can promote cooperation, but dedicators’ willingness to donate is more important than the amount of money they donate. As we can see in Fig 6a and 6b, when λ = 0, the blue curve drops down to the bottom as time goes by, which means *Ts* and *ρ* are getting closer to 0. While λ > 0, corresponding curves keep on high level, which means *Ts* and *ρ* have relatively large value. Similar results can be found in Fig 6c. It is clear that when λ is 0, both curves are close to the bottom. When λ changes from 0.2 to 2, the red and blue curves maintain high level steadily, and *Ts* reach the required level. Based on the above results, we can sum up that once dedicators donate, they will promote cooperation and variation of donation quantity has little effect to influence. Therefore, we conclude that dedicators’ willingness to donate is more important than the quantity to facilitate cooperation.

**Fig 7:**
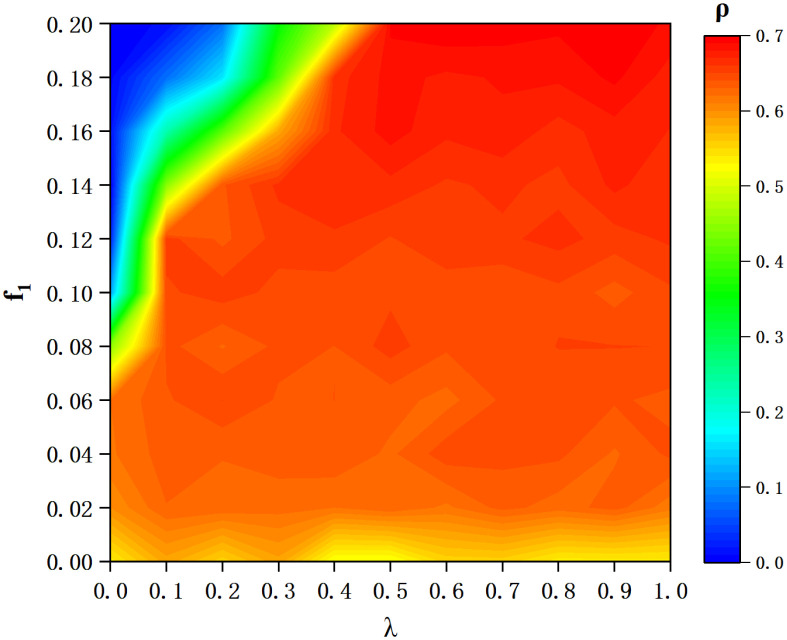
Heat-maps of cooperation frequency *ρ* at equilibrium along 2D plain based on proportion of dedicators *f*_1_ and the donation threshold of a dedicato λ. All kinds of colors represent various cooperation frequency under the joint action of different value of *f*_1_ and λ. The X-axis is λ (from 0 to 1) and the Y-axis is *f*_1_ (from 0 to 0.2).

As we know, *f*_1_ and λ are the significant factors for dedicator’s monetary contributions *C*_*d*_. The upper left corner of the Heat-maps in Fig 7 where minimum of *ρ* appears is blue and green, which means that the cooperation rate is less than 50 percent. So, the combination of a large *f*_1_ and a small λ is not good for cooperation. The rest of the graph is mostly red and small areas of yellow, indicating that the combination of *f*_1_ and λ in these cases is favorable for cooperation. And in the red area, with the increase of *f*_1_, the color is gradually deepened and the level of cooperation is slowly improved.

**Fig 8:**
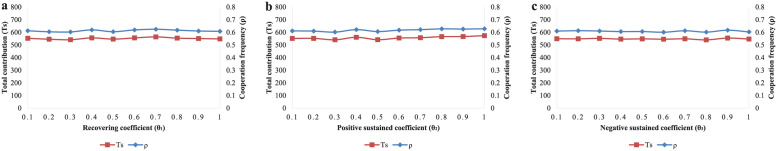
The equilibrium results of total contributions *Ts* and cooperation frequency *ρ* for different values of *θ*_1_, *θ*_2_ and *θ*_3_. (a)The equilibrium results of total contributions and cooperation frequency for different values of recovering coefficient *θ*_1_ varying from 0.1 to 1 with an interval of 0.1. (b) The equilibrium results of total contributions and cooperation frequency for different values of positive sustained coefficient *θ*_2_ varying from 0.1 to 1 with an interval of 0.1. (c)The equilibrium results of total contributions and cooperation frequency for different values of negative sustained coefficient *θ*_3_ varying from 0.1 to 1 with an interval of 0.1.

Next, recovering coefficient *θ*_1_, positive sustained coefficient *θ*_2_ and negative sustained coefficient *θ*_3_ are discussed in this part. It can be observed that curves in Fig 8a–8c, are almost horizontal. No matter how the values of *θ*_1_, *θ*_2_ and *θ*_3_ change, the blue and red curves always keep on stable high level. It means that the recovery speed of dedicators’ donation willingness, reduction velocity of emotional effect and the continuous influence of bad performance have little difference in impact on the evolution of cooperation. This finding is somewhat unexpected. It shows the model is so strong robustness that it can resist the impact of these three parameters.

**Fig 9:**
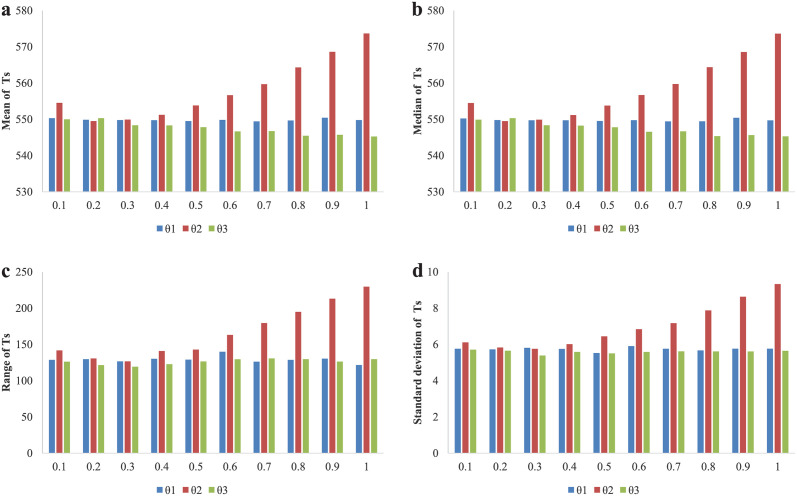
Results to show the mean, median, range and standard deviation of total contributions based on *θ*_1_, *θ*_2_ and *θ*_3_. (a)-(d) The result of mean, median, range and standard deviation of *Ts* for *θ*_1_, *θ*_2_ and *θ*_3_ varying from 0.1 to 1 with an interval of 0.1, respectively.

To analyze the robustness more detailly, we depict Fig 9. Compare to Fig 8, it can be seen that the mean and median values of *Ts* obtained based on *θ*_1_, *θ*_2_ and *θ*_3_ from Fig 9a and 9b are similar to the equilibrium results in Fig 8, all of which are around 550. Besides, Fig 9a–9d, we can see that the height of the blue and green columns are both almost unchangeable. Obviously, the height of the red columns shows an overall upward trend. As a whole, this model is more robustness against *θ*_1_ and *θ*_3_ than *θ*_2_. What is more, it can be found that when the value of *θ*_2_ is less than 0.5, the result has a little stronger stability.

**Fig 10:**
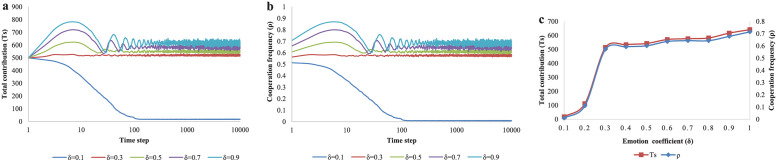
The evolution results of total contributions *Ts* and cooperation frequency *ρ* for different values of emotion coefficient *δ*. (a) The evolution process of total contributions for different values of emotion coefficient (*δ* = 0.1,0.3,0.5,0.7,0.9). (b)The evolution results of cooperation frequency for different values of emotion coefficient (*δ* = 0.1,0.3,0.5,0.7,0.9). (c)The equilibrium results of total contributions and cooperation frequency for different values of emotion coefficient *δ* varying from 0.1 to 1 with an interval of 0.1.

In addition to the parameters above, the attention is transferred to the emotion coefficient *δ*, which represents the emotional effect intensity of donating behavior of dedicators. We find that the greater *δ* is, the better cooperation level can get and there is a threshold (about 0.3 here) for *δ*. In Fig 10a and 10b, when *δ* = 0.1, the dark blue curve moves rapidly to a value close to 0. Once *δ* reaches or exceeds 0.3, the other curves eventually remain high level. We can clearly see in Fig 10c, as *δ* rises 0.1 to 1, the red and blue curves are always increasing. When *δ* is less than 0.3, the red and blue curves rise rapidly, but *Ts* and *ρ* are low. While *δ* rises from 0.3 to 1, the red and blue curves remain high level and increase slowly.

**Fig 11:**
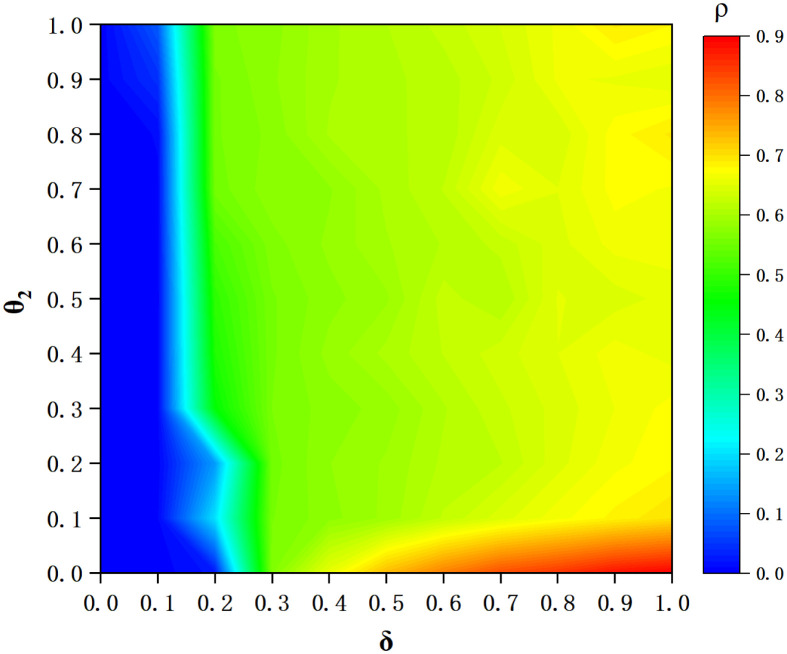
Heat-maps of cooperation frequency *ρ* at equilibrium along 2D plain based on positive sustained coefficient *θ*_2_ and emotion coefficient *δ*. All kinds of colors represent various cooperation frequency under the joint action of different *θ*_2_ and *δ*. The X-axis is *δ* (from 0 to 1) and the Y-axis is *θ*_2_ (from 0 to 1).

For further research, we plot the heat maps cooperation frequency *ρ* at equilibrium of *θ*_2_ and *δ*. *θ*_2_ and *δ* are the important parameters of extra cooperation probability *ω*. In Fig 11, we find that the closer to the right of the image, the brighter it is. This also means that the larger *δ*, the higher *ρ*. The maximum value of *ρ* appears in the lower right corner of the image, and a state close to all-C is obtained when *θ*_2_ < 0.1 and *δ* > 0.4. When *δ* < 0.1, a state close to all-D appears.

**Fig 12:**
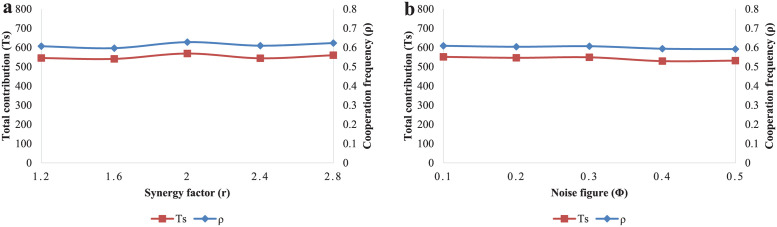
The equilibrium results of total contributions *Ts* and cooperation frequency *ρ*. (a) The equilibrium results of total contributions and cooperation frequency for different synergy factors (*r* = 1.2, 1.6, 2, 2.4, 2.8). (b) The equilibrium results of total contributions and cooperation frequency for different noise figure (*ϕ* = 0.1, 0.2, 0.3, 0.4, 0.5).

Finally, the robustness analysis of synergy factor *r* and noise figure *ϕ* will be discussed in the following content. As shown in Fig 12a, two curves run smoothly while the value of *r* changes from 1.2 to 2.8. Generally speaking, the cooperation level will not increase remarkably, as the value of synergy factor rises in a moderate interval. Then the effect of noise figure *ϕ* is described. In Fig 12b, *Ts* and *ρ* do not show significant fluctuation with different values of *ϕ*. It means the equilibrium results are robust against the parameter of *ϕ* in our PGG model.

## Conclusion

The government of China is focusing on providing satisfactory public cultural services. A series of measures has been taken to attract the public to participate in public cultural construction to easing the shortage of local government. As a successful spontaneous activity, Yunlin Gala provides a good case to research the origin and stability of cooperation in public cultural activities. The difference of population structure is an important distinction between activities in real social life. Differing from the common cooperator and defector, the dedicator is a typical example, who focuses on the group goal more than personal one. Based on the mechanisms of emotions, the role of dedicators on the evolution of cooperation is investigated in this paper. Through massive simulations, the specific conclusions are shown in the following: Firstly, by our researching, we find that the presence of dedicators can promote the cooperation in PGG. Then, a moderate budget of the activity is significant. A too high budget will lead to a collapse of the whole cooperation system. Next, dedicators’ willingness to donate is more important than their donation quantity in facilitating cooperation. Moreover, the stronger the emotional effect intensity of dedicators’ donating behavior is, the better. So, the unselfish donations of dedicators should be appreciated publicly to improve the emotional effect intensity. This is according with the reality, because the People Who Moved Yunlin Award which is set up to speak highly of donating dedicators has been existed for several years. We think it plays a positive role on promoting cooperation in the public culture activities.

Our study can make some contributions to policy makers and managers. For one thing, public cultural activities should be conducted by those people who pay more attention to group goals. For another, activity budget should be within the scope of the local financial capacity. What’s more, the selfless dedication of participants should be praised to promote cooperation by improving the emotional effect intensity.

In summary, we have investigated the influence of dedicators on evolution of cooperation in PGG. We find that dedicators can help avoid the tragedy of the commons by stabilizing and promoting cooperation. We hope that this study could shed a light into the understanding of the origin and stability of cooperation.
